# Does the Type of Cancer Influence Anti-Müllerian Hormone Levels in Women of Reproductive Age? A Cross-Sectional Study

**DOI:** 10.3390/biomedicines13102542

**Published:** 2025-10-18

**Authors:** Anna Maria Caringella, Antonio Stanziano, Clementina Cantatore, Angela Vitti, Anna Cortone, Antonio D’Amato, Raffaele Tinelli, Carmen Imma Aquino, Alessandro Libretti, Giuseppe D’Amato

**Affiliations:** 1 Department of Advanced Reproductive Risk Management and High-Risk Pregnancies, Reproductive and IVF Unit, PTA Conversano, ASL Bari, Via Edmondo De Amicis, 16, 70124 Bari, Italy; a.caringella@libero.it (A.M.C.); dottantoniostanziano@gmail.com (A.S.); clemecant@yahoo.it (C.C.); angelavitti@hotmail.it (A.V.); cortone.anna@gmail.com (A.C.); g.damato@iol.it (G.D.); 2 Obstetrics and Gynecology Unit, Department of Biomedical Sciences and Human Oncology, University of Bari “Aldo Moro”, Piazza Giulio Cesare 11, 70124 Bari, Italy; antoniodamato19@libero.it; 3 Department of Obstetrics and Gynecology, “Valle d’Itria” Hospital, 74015 Martina Franca, Italy; 4 Department of Gynecology and Obstetrics, University Hospital Maggiore Della Carità, Corso Mazzini, 18, 28100 Novara, Italy; c.immaquino@gmail.com

**Keywords:** anti-Müllerian hormone (AMH), ovarian reserve, fertility preservation, cancer, reproductive age

## Abstract

**Background:** Cancer treatments can damage the ovaries, with implications for fertility and reproductive lifespan. Therefore, a useful biomarker for fertility preservation counseling is needed, and anti-Müllerian hormone (AMH) measurement provides an index of the treatment gonadotoxicity. The debate is currently open as to whether the ovarian reserve may already be reduced before exposure to anticancer therapy. Therefore, our aim was to evaluate the influence of cancer on AMH levels. **Methods:** The present retrospective, cross-sectional study was carried out at the Centre for Reproductive Medicine and IVF Unit in Conversano, ASL Bari (Bari, Italy). All data were collected between 2019 and 2023. The serum AMH levels of 175 female patients with cancer were compared with those of non-cancer patients of reproductive age, just before starting chemotherapy. **Results:** AMH levels in women with breast cancer did not differ significantly from those in women without breast cancer (2.83 [0.81–9.15] ng/mL vs. 2.58 [0.7–9.2] ng/mL; *p*-value = 0.23). The AMH levels of the non-Hodgkin or Hodgkin lymphoma group were significantly lower than those of the non-cancer group (1.9 [0.7–7.0] vs. 3.2 [0.9–10.00] ng/mL; *p*-value < 0.05). **Conclusions:** AMH levels of non-Hodgkin or Hodgkin lymphoma patients were already reduced before cancer therapy compared to those of non-cancer patients. These results may be related to the systemic effect of the lymphoma, compared with other types of cancer.

## 1. Introduction

The preservation of fertility in cancer patients is an important consideration in its treatment [1]. Worldwide, the incidence of cancer in women aged 15–39 years is 48.7/100,000, and in women aged 40–44 years, it is 180.1/100,000, representing 13% of all newly diagnosed neoplasms [2].

Exposure to cancer treatments such as chemotherapy or radiotherapy can cause a decline in the reproductive function.

Cancer treatments can damage the ovaries, with implications for future fertility and reproductive lifespan. It has been estimated that approximately 68% of cancer survivors experience amenorrhea, a reduction in ovarian reserve, infertility, and, finally, premature ovarian failure (POI) [3,4].

The American Society of Clinical Oncology (ASCO) has published guidelines recommending that oncologists discuss with women the possibility of infertility resulting from treatment during their reproductive years [5].

The American Society for Reproductive Medicine (ASRM) and the European Society of Human Reproduction and Embryology (ESHRE) have strongly recommended providing a consultation on fertility preservation prior to cancer therapy [5,6,7]. Additionally, chemoprotection with gonadotropin-releasing hormone agonists after oocyte cryopreservation for mitigating the damage to ovarian reserve is a subject of debate in the living literature [7,8].

Measurement of anti-Müllerian hormone (AMH), produced by granulosa cells of growing follicles, is widely used as a marker of ovarian reserve and has a potential as a diagnostic and predictive biomarker of premature ovarian insufficiency (POI) [9]. Furthermore, AMH measurement provides an index of treatment gonadotoxicity, allowing the comparison of different medical regimens, although the assessment of medication effects on fertility requires caution (9). There is an ongoing debate as to whether the ovarian reserve in cancer patients may already be reduced before exposure to anticancer therapies.

Therefore, it is important to determine whether the ovarian reserve in cancer patients is reduced before or after cancer therapy.

Previous studies have investigated the ovarian reserve markers in cancer patients before the initiation of gonadotoxic treatments, but their results have often been inconsistent. In women with breast cancer, several studies have reported AMH levels comparable to age-matched healthy controls, suggesting that the disease itself may not impair the ovarian reserve prior to the therapy [1,2,3,4]. Conversely, other studies have observed lower AMH levels in this population, possibly related to the disease stage or other systemic factors [3,6]. Data on patients with lymphoma are even more heterogeneous: some reports have described reduced AMH levels already at diagnosis [5,6,7], whereas others found no significant differences compared with controls [1,2,3,4,5,6,7,8]. For other malignancies, available evidence is scarce and often limited by small sample sizes. Overall, the impact of cancer type on baseline ovarian reserve remains incompletely understood, with limited comparative data across tumor types.

The aim of this study was to evaluate the influence of cancer on serum anti-Müllerian hormone (AMH) levels.

## 2. Materials and Methods

We retrospectively reviewed the data retrieved from the Institutional Database in our Reproductive and IVF Unit, Department of Advanced Reproductive Risk Management and High-Risk Pregnancies, ASL Bari, Bari, Italy.

We retrieved and reviewed the records of women treated between January 2018 and December 2022.

For this retrospective, observational, cross-sectional study, our aim was to compare the AMH levels of reproductive age cancer-affected patients, before starting chemotherapy, in respect to an age-matched group of non-cancer patients. Inclusion criteria for the analysis were (a) cancer and non-cancer patients aged 18–40 years, with no history of therapy (chemotherapy/radiation), (b) cancer and non-cancer patients with no history of previous IVF treatment, and (c) non-cancer patients attempting the first IVF treatment for the male factor. Cancer and non-cancer patients with endometriosis were excluded for the analysis. Patients with polycystic ovary syndrome (PCOS), diagnosed according to the Rotterdam criteria, were excluded from both the cancer and control groups, as increased AMH levels in this population may lead to a misinterpretation of the ovarian reserve.

Serum AMH levels were measured from a serum sample during the first consultation and before starting chemotherapy, using Elecsys ^®^AMH (Elecsys^®^ AMH Anti-Müllerian Hormone, Roche Diagnostics, Indianapolis, IN, USA).

The test is based on the reaction of AMH with immuno-antibodies (monoclonal AMH-specific antibodies) and chemo-luminescence measurements of the reaction products. It consists of two incubation steps and a chemiluminescent measurement obtained with a photomultiplier tube (Elecsys^®^ AMH Anti-Müllerian Hormone, Roche Diagnostics, Indianapolis, IN, USA).

Antral follicle count (AFC) was measured with transvaginal ultrasound by experienced operators according to the current guidelines and clinical practice.

All procedures were conducted in accordance with the ethical standards of the institutional and/or national research committee and with the Declaration of Helsinki of 1964 and its subsequent amendments or equivalent ethical standards. The design, analysis, interpretation of data, writing, and revisions are conformed to the guidelines of the Committee on Publication Ethics (COPE) (http://publicationethics.org, accessed on 10 October 2025) and the RECORD (reporting of studies conducted using observational routinely collected health data) statement [10], available through the EQUATOR (enhancing the quality and transparency of health research) network (www.equator-network.org, accessed on 10 October 2025). This study was not advertised, and no compensation was offered to encourage patients to consent to the collection and analysis of their data. Written informed consent for all procedures and for data collection and analysis was obtained from all participants included in this study. The control group consisted of healthy women of reproductive age who attended our center for fertility assessment during the study period. They were not randomized; instead, they were selected consecutively and matched for age to the cancer patients to ensure comparability. All data were collected prospectively as part of routine clinical care.

This is a retrospective, observational, non-interventional study. Data were collected anonymously, and informed consent was obtained from all the enrolled patients. In view of this, the local ethics committee waived the requirement for ethical approval.

### 2.1. Ovarian Stimulation Protocol

In cancer patients’ group (non-Hodgkin/Hodgkin lymphoma patients), ovarian stimulation was performed using a GnRh antagonist protocol and administration of recombinant-FSH, at the starting dose of 150 IU–450 IU per day according to the patient’s age and ovarian reserve markers.

Since fertility preservation often requires emergency treatment, ovarian stimulation in this group was randomly started during the early follicular, late follicular, or luteal phase of the cycle as determined by serum progesterone levels and ultrasound evaluation (random start protocol).

The dose of gonadotropins was adjusted according to the ovarian response, serial ultrasound examination, and serum routine hormonal measurement (follicular-stimulating hormone, FSH; luteinizing hormone, LH; estradiol, E2; progesterone, P) every two days. The administration of GnRH antagonist was started when the leading follicle size reached 14 mm.

When two leading follicles reached 17–18 mm diameter, ovulation was triggered by human chorionic gonadotropin (hCG) 10.000 IU s.c. (Gonasi©, IBSA, Lodi, Italy) followed by oocyte retrieval 35–36 h later. In case of a high ovarian response or double stimulation, ovulation induction was carried out by administering triptorelin 0.2 mg (Decapeptyl^®^; Ferring Pharmaceuticals Korea Co., Ltd. 14th floor, Korea AD Culture Center, Olympic-ro 35-137, Songpa-gu, Seoul 138-921, Korea).

In breast cancer patients, ovarian stimulation was conducted using the letrozole-combined GnRH antagonist protocol on the day of the initial visit, without consideration of the menstrual cycle day (random start protocol). Ovarian stimulation was performed with recombinant follicle-stimulating hormone. The starting dose of gonadotropin was administered according to the ovarian reserve and the patient’s age. When the leading follicle reached a mean diameter of 13–14 mm, a GnRH antagonist was added to inhibit a premature LH surge. When two or more leading follicles reached a mean diameter of ≥20 mm, the final oocyte maturation was triggered using one of the following triggering GnRH agonists (0.2 mg of triptorelin; Decapeptyl^®^; Ferring Korea). Five milligrams of letrozole (Novartis, Switzerland) were co-administered with gonadotropin from the start of the cycle and continued until the trigger day. Letrozole was skipped the day after the trigger and resumed after oocyte retrieval. The drug was continued for at least one week until the serum estradiol (E2) levels remained lower than 50 pg/mL. 

Oocyte retrieval was performed by a transvaginal sonography-guided technique 35–36 h after the triggering of the ovulation.

Mature oocytes (MII), documented by the presence of one polar body, were vitrified as previously described using a ^®^Kitazato Vitritification Kit (Kitazato Corporation, 100-10 Yanagishima, Fuji, Shizuoka 416-0932 Japan).

### 2.2. Statistical Analysis

Statistical analysis was completed by using SPSS Statistics, version 22. All data were presented as means ± standard deviation (SD), with the range in square brackets or a number (percentage). Comparisons between normally distributed continuous variables were made by using Student’s *t*-test. Comparisons between categorical variables were made by using contingency tables and the Chi-Squared test. A value of *p* < 0.05 was considered as statistically significant. No formal adjustments for multiple comparisons were performed; therefore, the results should be interpreted as exploratory.

No formal sample size calculation or power analysis was performed prior to this study. The sample size corresponds to all eligible patients attending our fertility preservation program during the study period.

## 3. Results

During the study period, we collected data from 175 female patients on the reproductive age before starting chemotherapy for cancer. The median age was 31 years (18–40): 79 women had a diagnosis of breast cancer, 63 non-Hodgkin or Hodgkin lymphoma, and 33 women other cancer (gynecological cancer, rectal cancer, tongue cancer, nasopharyngeal cancer, myelodysplastic syndrome, bone cancer). The distribution of cases is shown in Table 1.

Given the small sample size and the heterogeneity of tumor types within this group, no separate statistical analysis was performed for these patients. Therefore, detailed comparisons with the other groups were not conducted.

We mainly focused our analysis on breast cancer and or Hodgkin/non-Hodgkin lymphoma patients. The AMH levels of cancer patients prior to chemotherapy were compared to those of a matched group of non-cancer patients on reproductive age.

Regarding breast cancer patients, the baseline characteristics between the two groups were homogenous in terms of age, body mass index (BMI), age at menarche, oral contraception, cigarette smoking, and parity (*p* > 0.05) (Table 2). The antral follicle count (AFC) was comparable between the two groups (*p* > 0.05), and the AMH levels in the breast cancer group were found not to be significantly different to those in the non-cancer group (2.83 [0.81–9.15] ng/mL vs. 2.58 [0.7–9.2]; *p*-value =0.23).

Regarding the non-Hodgkin/Hodgkin lymphoma, the baseline characteristics of the two groups were comparable with age, BMI, age of menarche, oral contraception, cigarette smoking, and parity (*p* > 0.05) (Table 3). The AMH levels of non-Hodgkin or Hodgkin lymphoma group were significantly lower in comparison to those in the non-cancer group (1.9 [0.7–7] vs. 3.2 [0.9–10.00] ng/mL; *p*-value < 0.05); on the other side, the AFC of the cancer group was found to be not significantly different from that in the non-cancer group.

We compared baseline characteristics and AMH levels of non-Hodgkin/Hodgkin lymphoma with patients diagnosed with breast cancer (Table 4). We observed that the AMH levels of the non-Hodgkin or Hodgkin lymphoma group were significantly lower in comparison to those in the breast cancer group; on the other side, AFC was found to be not significantly different from those in the other two groups.

We compared ovarian stimulation cycle results of non-Hodgkin/Hodgkin lymphoma with breast cancer patients (Table 4).

We found a mean of 7.5 [2–19] total oocytes retrieved, with a mean of 6.7 [2–19] MII oocytes in lymphoma patients’ group, and a mean of 9.1 [5–22] total oocytes retrieved and of 8.3 [5–22] in the breast cancer patients’ group (Table 4).

## 4. Discussion

Advances in cancer therapy have entailed an increase in the survival rate [7]. Anticancer treatments can affect the ovarian reserve by a mechanism of inducing a depletion of growing and primordial follicles. The decrease in the ovarian reserve can shorten the reproductive lifespan, leading to POI [9,11]. This phenomenon has significant implications for women’s health and is linked to infertility or other clinical disorders such as cardiovascular disease, neurological disease, and osteoporosis [9,10,11,12,13,14]. Consequently, prior to initiating treatments that increase the risk of POI, decision-making regarding the choice of anticancer treatment and additional fertility preservation interventions should be considered.

The measurement of circulating AMH, produced by granulosa cells of growing follicles, is widely used to evaluate the ovarian reserve. This hormone is a potential diagnostic and predictive biomarker of the ovarian function [9]. Indeed, AMH levels are often significantly impaired by anticancer treatment and show a highly variable recovery rate [1]. Nevertheless, the reliability of AMH in evaluating the ovarian reserve in different types of cancers is still debated.

Several studies have shown that AMH levels are lower in patients with lymphoma even before the initiation of treatment [1,15,16]. These findings suggest that the disease itself may have an impact on the ovarian reserve, independently from chemotherapy. However, data across cancer types remain heterogeneous, and the overall evidence is limited.

As we know, the recurrence of several types of cancers, such as Hodgkin’s and non-Hodgkin’s lymphoma and breast cancer, may be associated with decreased ovarian reserves before cancer therapy. Lawrenz et al. found that the AMH levels of lymphoma patients were lower than those of non-cancer patients, with mean AMH levels of 2.06 ng/mL vs. 3.20 ng/dL (*p*-value < 0.05) [15]. In the same way, Lekovich J et al. [16] and Yu B et al. reported AMH levels in breast cancer patients not reduced compared with controls [17].

Su et al. carried out a similar study to assess AMH levels in breast cancer patients and found that they were lower than in the non-cancer patients (0.6 ng/mL vs. 1.1 ng/mL, *p*-value < 0.001) [18]. In addition, a study comparing pre-treatment AMH levels in young cancer patients (<18 years) with age-matched non-cancer patients was conducted by Van et al. in 2014 [19], where AMH levels were found to be significantly lower in cancer patients [19]. The results of these studies were consistent with the findings from other studies [3,20,21,22]. Another study found that 44 cancer patients of reproductive age had lower pre-treatment AMH levels than non-cancer patients, with a mean of 1.11 [0.08–4.65] ng/mL versus 3.99 [1.19–8.7] ng/mL (*p*-value < 0.001) [3]. A summary of the results of these studies is shown in Table 5.

In our study, we collected data from 79 patients diagnosed with breast cancer and 63 patients diagnosed with non-Hodgkin’s or Hodgkin’s lymphoma, respectively. The AMH levels of the breast cancer group were found not to be significantly different compared with those in the non-cancer group. On the other hand, the AMH levels of the non-Hodgkin’s or Hodgkin’s lymphoma group were found to be significantly lower compared to those in the non-cancer group and in the breast cancer group.

A complete knowledge of factors affecting AMH levels is still elusive, but these may be influenced by genetic and environmental factors. Ovarian aging, defined as a gradual decrease in both the quantity and quality of the oocyte production and maturation, occurs during women’s life. This decrease is linked to both chronological and biological age [20]. Chronological age is determined by the time since birth, while biological age is determined by physiology [21]. Biological age has a greater impact on reproductive function than chronological age. Furthermore, the ovarian reserve represents a good marker of biological age. Some studies have shown that the biological age of the cancer group was 10 years higher compared to the non-cancer group, indicating that ovarian aging occurs earlier in patients diagnosed with cancer [3]. Regarding environmental factors, Su et al. found that age, BMI, parity, and smoking status were not associated with decreased levels of AMH in patients with breast cancer [18]. However, the different results between the studies may be due to different environmental conditions and the heterogeneity of the populations studied. In the context of breast cancer, the mutations of BRCA1 and BRCA2 genes could exert an important impact on the ovarian function [1]. These genes encode proteins that are involved in the DNA damage repair pathway [22]. There is growing evidence from animal models that BRCA1 plays a crucial role in preserving the fertility and extending the ovarian lifespan [23]. Studies have also shown that women with BRCA1 mutations have lower AMH levels and a reduced response to ovarian stimulation [23]. The BRCA1/2 carriage has also been linked to an earlier onset of menopause [24]. Women with BRCA1 mutations tend to have a lower AMH level overall. In a study including 172 patients carrying a BRCA1 mutation, it was estimated to be 25% lower [24,25]. Johnson et al. [25] observed that BRCA2 mutation carriers had significantly lower AMH levels compared to controls. As a tumor suppressor gene, BRCA2 is involved in regulating the follicular pool through impairment of the DNA repair pathway and can affect ovarian reserves in breast cancer patients. In our study, regarding BRCA status, we observed that 6 patients carried a BRCA mutation: 4 BRCA 1 and 2 BRCA2; 8 patients were negative for BRCA mutations; and 65 women had an unknown BRCA status. However, given the small number of patients with known BRCA mutations (n = 6) and the large proportion with an unknown status, any conclusions regarding the impact of BRCA mutations on the ovarian reserve should be interpreted with caution. This limited sample size reduces the generalizability of BRCA-related findings in our cohort.

Previous studies have shown that breast cancer patients carrying BRCA mutations may present with reduced AMH levels even before the initiation of gonadotoxic therapy, and the BRCA status has been identified as an important parameter in predicting the long-term ovarian function. Although our sample size was too small to allow a meaningful analysis, this aspect is clinically relevant and should be further investigated in larger cohorts.

Reduced AMH levels in Hodgkin/non-Hodgkin lymphoma patients prior to the start cancer therapy can be related with the systemic effect of lymphoma compared to other kinds of cancers. In fact, high cytokines in lymphoma patients indirectly affect the ovarian reserve [26,27]. Few studies on lymphoma patients are available, and those that are show significantly lower AMH levels than in the control group. There is a strong negative correlation between AMH levels and SIL-2R, IL-6, and IL-8 cytokine concentrations [26].

In our cohort, inflammatory markers or cytokine profiles were not measured. Therefore, this hypothesis could not be directly tested and remains speculative.

DNA repair mechanism impairment may also contribute to reduced AMH levels in childhood cancer cases [19].

AMH has been recognized as a good marker for measuring the ovarian follicular reserve in women undergoing chemotherapy. Its longitudinal variations can discriminate between highly and minimally toxic chemotherapy protocols affecting ovarian function. It has been shown in various types of cancers that age, chemotherapy regimen, and pre-treatment AMH levels are the main predictors of the ovarian recovery function. A systematic review conducted by Anderson et al. [9] including 9183 patients evaluated AMH as a biomarker of the ovarian reserve and the likelihood of POI recurrence before and after cancer treatment. The authors confirmed that cancer treatment has a substantial impact on AMH levels, leading to a follicular depletion during treatment, followed by a period of partial recovery peaking within 1–2 years. Low post-treatment AMH was predicted by lower pre-treatment AMH, according to age and treatment gonadotoxicity. Low post-treatment AMH correlated with an increased likelihood of POI. However, there were very limited data relating AMH to fertility or relating time to age at POI or menopause. Therefore, AMH is a useful biomarker of the ovarian reserve before and after cancer treatment, and in the diagnosis of POI after cancer treatment, but the relationships between post-treatment fertility and reproductive lifespan require further investigation [9].

The value of AMH at the time of diagnosis and in terms of ovarian function has clearly been demonstrated in women diagnosed with breast cancer, but limited data are available for women with other cancer types. Large studies on longitudinal AMH variations under chemotherapy in patients diagnosed with lymphoma are few but they provide the opportunity to assess the degree of follicular loss at a young age. Decanter et al. [28] conducted a prospective cohort study on non-Hodgkin or Hodgkin lymphoma patients aged between 15 and 35 years who were recruited before chemotherapy. Patients were treated with either a non-alkylating protocol or by an alkylating regimen. The authors concluded that the pre-treatment AMH levels influenced the pattern of the AMH variation both in alkylating and ABVD protocols. Likewise, age was significantly associated with the pattern of the recovery phase only in the alkylating group. BMI had no influence on the AMH recovery phase whatever the protocol.

In women with breast cancer, the pre-treatment AMH level predicts long-term ovarian function measured as ongoing menses recurrence [1]. In their first studies, Andersen et al. [29,30] demonstrated that AMH was a better predictor than age, although this latter variable is also an important predictive factor. In a second similar prospective cohort study, it was shown that pre-treatment AMH below the median value of 0.46 ng/mL accurately predicted amenorrhea in all women, two years following the diagnosis [31]. Nevertheless, in different cancer survivors, it has been shown that pre-treatment AMH levels impact on the recovery rate after chemotherapy. Higher pre-treatment AMH levels were associated with more rapid resumption of the ovarian function [32]. AMH levels correlate well with other markers of the ovarian reserve, such as antral follicle counts and measurements of inhibin B [33]. In our study, we observed that the AFC of the cancer group was not significantly different compared to that in the non-cancer group. The same results were obtained when comparing the breast cancer patients with the non-Hodgkin’s or Hodgkin’s lymphoma group. The cytokines released by the lymphoma may have a detrimental effect on the AMH production and thus on the AMH blood concentration despite an adequate count of AFC. Currently published studies have consistently shown that both AFC and serum AMH are good predictors of ovarian response, and they have shown strong correlations. However, discordance between them is described. As a direct product of granulosa cells from preantral and small antral follicles during the early follicle maturation process, AMH indirectly reflects the primordial follicle pool. In comparison, AFC comprises the number of 2–10 mm diameter follicles that can be visualized by ultrasound; hence, AMH reflects an additional population of preantral follicles, thus serving as a better proxy of oocyte supply. Moreover, it is worth noting that ultrasound technology cannot distinguish healthy from atretic follicles, which may hinder AMH production. In addition to these, both indicators can be influenced by comparable technical, physiological, and exogenous factors. Furthermore, in our study, AFC in control group was measured on days 2–5 of the spontaneous menstrual cycle, while in cancer patients, it was measured randomly since fertility preservation often requires emergency treatment.

This methodological difference is important, as AFC measured outside the early follicular phase may not accurately reflect the true antral follicle pool. This could partly explain the observed discordance between AFC and AMH levels in our cohort

The level of AMH has been found to be correlated with the oocyte number collected during the ovarian stimulation in the fertility preservation program [34]. Overall, the results of ovarian stimulation are similar in women with cancer compared to women without cancer [35].

However, there is good evidence of a reduced oocyte quality in women with cancer compared to those undergoing a social freezing program, which is not reflected by AMH levels [36,37].

Lawrenz B et al. [15] analyzed the number of oocytes retrieved in 64 lymphoma and 84 breast cancer patients, and the results from the lymphoma patients’ group were compared with those of the breast cancer patients. The authors reported a mean of 11.62 ± 6.21 oocytes retrieved in lymphoma patients’ group and a mean of 12.10± 8.37 oocytes retrieved in breast cancer patients.

Lekovich J et al. [16] showed that patients in the lymphoma group demonstrated significantly lower AMH levels and AFC and had less oocytes harvested and cryopreserved when compared to healthy controls (social freezing program).

A summary of the results of these studies is shown in Table 6.

In our study, we analyzed the number of oocytes retrieved in 63 lymphoma and 79 breast cancer patients, and the results from the lymphoma patients were compared with those of the breast cancer patients. We observed that the MII oocyte number in non-Hodgkin or Hodgkin lymphoma groups was significantly lower in comparison to those in the breast cancer group; on the other side, total oocytes retrieved were found not to be significantly different from those in the other two groups (Table 4).

Interestingly, in our study, although the AFC did not differ between the two groups, the AMH concentration was significantly lower in the lymphoma group compared to the breast cancer group, as well as MII oocyte number.

The levels of AMH in gynecological cancers were not discussed in our study. This is because local treatment of cancers of the genital organs can impair the reproductive function and affect the results of the analysis. Similarly, due to the heterogeneity and the limited number of cases included in the ‘other cancers’ groups, no separate analysis was performed. This represents a limitation of the present study, and future investigations with larger and more homogeneous subgroups are needed to better understand the impact of these cancer types on the ovarian reserve. Nevertheless, in other studies [15,16,17,18,19], the AMH levels of gynecologic patients of reproductive age prior to receiving cancer treatment were compared to those of non-gynecological cancer patients, and no significant difference was found. However, the authors concluded that the unequal size of the gynecological and non-gynecological cancer subject groups in the study may have affected the results [3]. There are several limitations of this retrospective study. It is important to recognize that controls were recruited from healthy women referred to the center for the IVF cycle due to male infertility, and we were unable to compare stimulation outcomes between cancer and non-cancer patients. For a future analysis, the control group should be composed of women enrolled for social freezing and/or egg donation programs. Moreover, baseline FSH and estradiol levels were not systematically available for all patients, particularly in the cancer group, as fertility preservation often required urgent intervention. This represents another limitation of this study. Finally, another limitation of this study was that we were unable to verify the correlations of AMH and age, genetic factors (BRCA status), and the stage of cancer due to the small sizes of the cancer groups.

For this reason, more data are required in future studies.

## 5. Conclusions

In conclusion, our data have shown that the levels of AMH are lower in women with lymphoma at the time of diagnosis compared with the levels in age-matched controls. In contrast, women with breast cancer do not appear to have reduced AMH levels. Furthermore, AMH levels and MII oocyte numbers are lower in women with lymphoma than in breast cancer patients. It could be speculated that this aspect may be due to the systemic inflammatory nature of the lymphoma in comparison to other types of cancers.

This highlights the importance of offering fertility preservation programs prior to cancer treatments and the need for a useful biomarker for fertility preservation counseling.

## Figures and Tables

**Table 1:** Cancer distribution.

Cancer	No. Cases (% ^1^)
Breast cancer	79 (45%)
Hodgkin/non-Hodgkin lymphoma	63 (36%)
Other cancers	33 (19%)
Total	175

^1^ Data were presented as number (percentage).

**Table 2:** Baseline characteristics and AMH levels of breast cancer and non-cancer group.

Characteristics	Cancer Group (n° = 79) [IQR]	Non-Cancer Group (n° = 79)	*p*-Value
**Age (Years)**	34 (±2.34) [27–40]	34.13 (±1.1) [27–40]	n.s. ^1^
**Menarche**	12 [10–14]	12 [10–14]	n.s.
**BMI**	22.3 (±3.0)	22.0 (±2.9)	n.s.
**Parity**	0	0	n.s.
**Oral contraception (>6 months) (%)**	18 (22)	12 (15)	n.s.
**Cigarette smoking (%)**	4 (5)	5 (6)	n.s.
**Cancer stage (%)**			
**0**	3 (4)		
**I**	24 (30)		
**II**	37 (46)		
**III**	15 (20)		
**IV**	0		
**BRCA status (%)**			
**BRCA 1+**	4 (5)		
**BRAC 2+**	2 (3)		
**Negative**	8 (10)		
**Unknown**	65 (82)		
**AFC**	10.5 [5–20]	11.3 (±3.16) [6–20]	n.s.
**AMH**	2.83 [0.81–9.15]	2.58 [0.7–9.2]	n.s.

^1^ n.s. non statistically significant.

**Table 3:** Baseline characteristics and AMH levels of non-Hodgkin/Hodgkin lymphoma and non-cancer group.

Characteristics	Cancer Group Non-Hodgkin/Hodgkin Lymphoma (n° = 63)	Non-Cancer Group (n° = 63)	*p*-Value
**Age, years (min–max)**	30.00 (±2.34) [24–40]	31.9 (± 2.59) [24–40]	n.s. ^1^
**Menarche**	12 [10–14]	12 [10–14]	n.s.
**BMI**	23.19 (±0.72) [18–31]	22.86 (0.83) [18–31]	n.s.
**Parity**	0	0	n.s.
**Oral contraception (>6 months) (%)**	14 (22)	12 (19)	n.s.
**Cigarette smoking (%)**	9 (14)	12 (19)	n.s.
**Cancer stage (%)**			
**I–II**	48 (76)		
**III–IV**	15 (14)		
**AFC**	10.4 [5–19]	11.1 (±2.04) [6–20]	n.s.
**AMH**	1.9 [0.7–7]	3.2 [0.9–10.00]	0.001

^1^ n.s. non statistically significant.

**Table 4:** Baseline characteristics, AMH levels, and ovarian stimulation outcomes of non-Hodgkin/Hodgkin lymphoma and breast cancer group.

Characteristics	Non-Hodgkin/Hodgkin Lymphoma (n° = 63)	Breast Cancer (n° = 79)	*p*-Value
**Age, years (min–max)**	30.00 (±2.34) [24–40]	34 (±2.34) [27–40]	n.s. ^1^
**Menarche**	12 [10–14]	12 [10–14]	n.s.
**BMI**	23.19 (±0.72) [18–31]	22.3 (±3.0)	n.s.
**Parity**	0	0	0
**AFC**	10.4 [5–19]	10.5 [5–20]	n.s.
**AMH**	1.9 [0.7–7]	2.83 [0.81–9.15]	0.03
**Stimulation length (days)**	11.8 [10–15]	11.2 [10–15]	n.s.
**COH start (%)**			
**Early follicular phase**	31 (49)	42 (53)	n.s.
**Late follicular phase**	10 (16)	17 (22)	n.s.
**Luteal phase**	22 (35)	20 (25)	n.s.
**Total units of gonadotropin (IU)**	1929 [1500–5325]	2152 [1500–5325]	n.s.
**Trigger (triptorelin)**	33/63	79/79	0.004
**Oocytes retrieved/cycle**	7.5 [2–19]	9.1 [5–22]	n.s.
**MII oocytes/cycle**	6.7 [2–19]	8.3 [5–22]	0.03

^1^ n.s. non statistically significant.

**Table 5:** Serum anti-Müllerian hormone (AMH) levels and cancer.

Authors	Year	Study Design	Type of Cancer	AMH Level (ng/mL)	
				Case	Age-Matched Controls
Lawrenz B et al. [15]	2012	Age-matched control study	Lymphoma	2.06 ± 1.52 *	3.20 ± 2.19 *
Lekovich J et al. [16]	2016	Retrospective study	Lymphoma	1.08 ± 0.74 *	2.03 ± 1.93 *
Yu B et al. [17]	2010	Nested prospective cohort study	Breast cancer	0.86 (0.07–9.1) ^	0.94 (0.2–7.7) ^
Su HI et al. [18]	2013	Cross-sectional study	Breast cancer	0.66 ± 3.6 *	1.1 ± 2.9 *
van Dorp W [19]	2014	Age-matched case–control study	Several cancers	1.4 (0.1–10.2) ^	3 (0.1–18.3) ^

* Values are expressed as mean ± SD. ^ Values are expressed as mean and range value.

**Table 6:** Anti-Müllerian hormone (AMH) levels, antral follicular count, total oocytes retrieved/cycle, and MII oocytes.

Authors	Type of Cancer	AMH Level (ng/mL)	AFC	Total Oocytes Retrieved/Cycle (n°)	MII Oocytes/Cycle (n°)
		Case	Case	Case	Case
Lawrenz B et al. [15]	64 lymphoma	2.06 ± 1.52 *	No data	11.62 ±6.21 *	No data
	84 breast cancer	No data	No data	12.10 ± 8.37 *	
Lekovich J et al. [16]	64 lymphoma	1.08 ± 0.74 *	9.41 ± 4.77	10.81 ± 6.49	8.12 ± 5.54
Yu B et al. [17]	Breast cancer	0.86 (0.07–9.1) ^	No data	No data	No data
Su HI et al. [18]	Breast cancer	0.66 ± 3.6 *	No data	No data	No data
van Dorp W [19]	Several cancers	1.4 (0.1–10.2) ^	No data	No data	No data
Our study	Lymphoma	1.9 [0.7–7]	10.4 [5–19]	7.5 [2–19]	6.7 [2–19]
	Breast cancer	2.83 [0.81–9.15]	10.5 [5–20]	9.1 [5–22]	8.3 [5–22]

* Values are expressed as mean ± SD. ^ Values are expressed as mean and range values.

## Data Availability

Data are available upon written request to the corresponding author.
